# Combining transcranial direct-current stimulation with gait training in patients with neurological disorders: a systematic review

**DOI:** 10.1186/s12984-019-0591-z

**Published:** 2019-09-14

**Authors:** Rubén Hernández de Paz, Diego Serrano-Muñoz, Soraya Pérez-Nombela, Elisabeth Bravo-Esteban, Juan Avendaño-Coy, Julio Gómez-Soriano

**Affiliations:** Toledo Physiotherapy Research Group (GIFTO), Faculty of Physiotherapy and Nursery, Castilla La Mancha University, 45071 Toledo, Spain

**Keywords:** Transcranial direct-current stimulation, Gait, Rehabilitation, Neurological disorder

## Abstract

**Background:**

Transcranial direct-current stimulation (tDCS) is an easy-to-apply, cheap, and safe technique capable of affecting cortical brain activity. However, its effectiveness has not been proven for many clinical applications.

**Objective:**

The aim of this systematic review was to determine whether the effect of different strategies for gait training in patients with neurological disorders can be enhanced by the combined application of tDCS compared to sham stimulation. Additionally, we attempted to record and analyze tDCS parameters to optimize its efficacy.

**Methods:**

A search in Pubmed, PEDro, and Cochrane databases was performed to find randomized clinical trials that combined tDCS with gait training. A chronological filter from 2010 to 2018 was applied and only studies with variables that quantified the gait function were included.

**Results:**

A total of 274 studies were found, of which 25 met the inclusion criteria. Of them, 17 were rejected based on exclusion criteria. Finally, 8 trials were evaluated that included 91 subjects with stroke, 57 suffering from Parkinson’s disease, and 39 with spinal cord injury. Four of the eight assessed studies did not report improved outcomes for any of its variables compared to the placebo treatment.

**Conclusions:**

There are no conclusive results that confirm that tDCS can enhance the effect of the different strategies for gait training. Further research for specific pathologies, with larger sample sizes and adequate follow-up periods, are required to optimize the existing protocols for applying tDCS.

## Introduction

Difficulty to walk is a key feature of neurological disorders [1], so much so that recovering and/or maintaining the patient’s walking ability has become one of the main aims of all neurorehabilitation programs [2]. Additionally, the loss of this ability is one of the most significant factors negatively impacting on the social and professional reintegration of neurological patients [3].

Strategies for gait rehabilitation traditionally focus on improving the residual ability to walk and compensation strategies. Over the last years, a new therapeutic paradigm has been established based on promoting neuroplasticity and motor learning, which has led to the development of different therapies employing treadmills and partial body-weight support, as well as robotic-assisted gait training [4]. Nevertheless, these new paradigms have not demonstrated superior results when compared to traditional therapies [5–7], and therefore recent studies advise combining therapies to enhance their therapeutic effect via greater activation of neuroplastic mechanisms [8].

Transcranial direct-current stimulation (tDCS) is an intervention for brain neuromodulation consisting of applying constant weak electric currents on the patient’s scalp in order to stimulate specific brain areas. The application of the anode (positive electrode) to the primary motor cortex causes an increase in neuron excitability whereas stimulation with the cathode (negative electrode) causes it to decrease [9].

The effectiveness of tDCS has been proven for treating certain pathologies such as depression, addictions, fibromyalgia, or chronic pain [10]. Also, tDCS has shown to improve precision and motor learning [11] in healthy volunteers. Improvements in the functionality of upper limbs and fine motor skills of the hand with paresis have been observed in patients with stroke using tDCS, although the results were somewhat controversial [12, 13]. Similarly, a Cochrane review on the effectiveness of tDCS in treating Parkinson’s disease highlights the great potential of the technique to improve motor skills, but the significance level of the evidence was not enough to clearly recommend it [14]. In terms of gait rehabilitation, current studies are scarce and controversial [10].

Furthermore, tDCS is useful not only as a therapy by itself but also in combination with other rehabilitation strategies to increase their therapeutic potential; in these cases, the subjects’ basal activity and the need for combining the stimulation with the behavior to be enhanced have been highlighted. Several studies have combined tDCS with different modalities of therapeutic exercising, such as aerobic exercise to increase the hypoalgesic effect in patients with fibromyalgia [15] or muscle strengthening to increase functionality in patients suffering from knee osteoarthritis [16]. Along these lines, various studies have combined tDCS with gait training in patients with neurological disorders, obtaining rather disparate outcomes [17–20]. As a result, the main aim of this systematic review was to determine whether the application of tDCS can enhance the effectiveness of other treatment strategies for gait training. Additionally, as a secondary objective, we attempted to record and identify the optimal parameters of the applied current since they are key factors for its effectiveness.

## Methods

### Search strategy

This systematic review conducted a search in three databases: PubMed, Physiotherapy Evidence Database (PEDro), and Cochrane controlled register of trials (CENTRAL). All searches were based on the same criteria and filtered the studies chronologically from 2010 to 2018. An inverse manual search was also performed from the trials found in the search.

The employed strategy included the following keywords: “Transcranial Direct Current Stimulation”, “tDCS”, “Gait”, “Walking”, and “Mobility training”, as well as their various combinations. The MeSH terms “Transcranial Direct Current Stimulation” and “Walking” were also entered in the PubMed and Cochrane databases search.

### Selection of studies

To select the studies, duplicates were firstly eliminated and a simple reading of titles and abstract of all found articles was carried out to discard those not complying with the established inclusion criteria. Articles passing this first filter were fully read in order to eliminate those meeting any of the exclusion criteria. The search and inclusion of studies in this review was carried out independently by two researchers (RHP and EBE), and no discrepancies were found between them. To assess the methodological quality of trials, the PEDro scale (Table 1) was used, whose reliability has been demonstrated [16].

**Table 1:** Methodological quality of included articles in accordance with the PEDro scale

	Geroin et al., 2011 [21]	Manji et al., 2018 [22]	Yotnuengnit et al., 2017 [23]	Chang, Kim, & Park., 2015 [24]	Raithatha et al., 2016 [25]	Seo et al., 2017 [26]	Costa-Ribeiro et al., 2017 [27]	Kumru et al., 2016 [28]
Eligibility criteria	√	√	√	√	√	√	√	
Random allocation	√	√	√	√	√	√	√	√
Concealed allocation			√			√	√	
Basal intergroup similarities		√	√	√		√	√	√
Blinding of participants	√	√	√	√	√	√	√	√
Blinding of therapists		√				√		
Blinding of assessors	√	√	√	√	√	√	√	√
Follow-up	√	√	√	√	√		√	√
Intention-to-treat analysis	√	√		√	√			√
Intergroup statistical comparison	√	√	√	√	√	√	√	√
Point measures and variability measures	√	√	√	√	√	√	√	√
Total score	7/10	9/10	8/10	8/10	7/10	8/10	8/10	8/10

### Criteria for inclusion and exclusion

Criteria for inclusion comprised randomized and controlled clinical trials published in English or Spanish. Subjects had to be diagnosed with a pathology of the central nervous system. At least one intervention group had to receive active tDCS applied in combination with a method of gait training, whether traditional rehabilitation, robotic-assisted rehabilitation, or a combination of both; also, the trials had to be controlled via a sham tDCS combined with gait training in a similar way as for the intervention group. The recorded variables had to quantify the gait, whether in a biomechanical, neurophysiological, functional, or clinical way.

Following the criteria for exclusion, the following articles were rejected: all those that did not record key parameters regarding the stimulation (intensity, placement of electrodes, and session duration); studies not including data on the duration of gait training, number of sessions, and rest intervals between them; using dual-task as treatment for gait rehabilitation due to a potential confusion factor; including subjects < 18 years of age; and using sham stimulation where the electrodes placement differed from that of the tDCS intervention group. Additionally, with the intention of adding clinical value to the assessed therapeutic programs, trials with less than five sessions and five included subjects in the stimulation group were discarded.

## Results

**Fig. 1:**
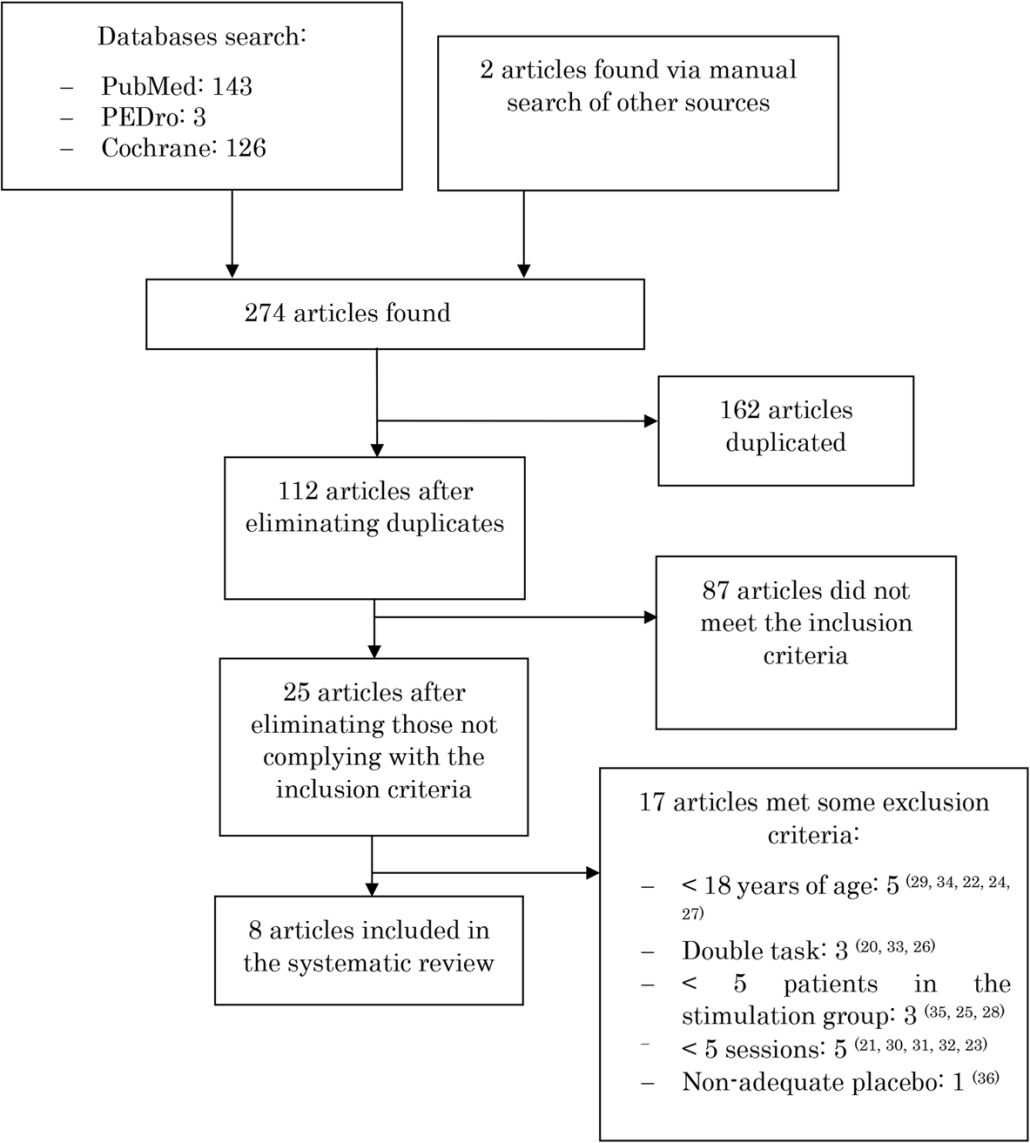
Flow of articles during the selection process

Of the 274 matches resulting from the search in the three databases (143 in Pubmed, 3 in PEDro, 126 in Cochrane, and 2 from a manual search in other sources), 162 articles were eliminated due to being duplicated, 87 did not meet the inclusion criteria, and 17 [25, 27–42] were rejected for meeting some of the exclusion criteria. Finally, eight articles were selected and included in this systematic review (Fig. 1). Table 2 shows the most relevant characteristics of the articles and their findings, which are discussed hereafter. None of the studies in this systematic review reported adverse or secondary effects for any intervention.

**Table 2:** Main characteristics and outcomes of the reviewed articles

Article	Pathology; N for the intervention group	Area of application and tDCS parameters (current density; duration)	Number of sessions; intervals	Design	Order of application; control group	Variables	Gait rehabilitation treatment; duration	Effect versus placebo (% difference, *p)*
Geroin et al., 2011 [23]	Stroke; *N* = 10.	A = PMI- affected LL, C = SO to-CL; DC: 0,04 mA/cm^2^; 7 min.	10 s; 2 weeks.	EV.1 = Pre EV.2 = Post EV.3 = 2 weeks post	ONLINE; Off since the beginning.	6MWT, 10MWT, BPG, FAC, RMI, MILS, MAS.	Exoskeleton robot; 20 min.	SNS
Manji et al., 2018 [24]	Stroke; *N* = 30. (crossover design)	A = 3.5 cm anterior to Cz (SMA), C = EOC; DC: 0.04 mA/cm^2^; 20 min.	7 s; 1 week (3-day washout period)	EV.1 = Pre EV.2 = Post	ONLINE; UNK.	10MWT, TUG, FMA, POMA, TIS.	Exoskeleton-robot; 20 min.	↓10MWT (9.09%, *p* = 0.046) and ↓TUG (5.29%, *p* = 0.026)
Yotnuengnit et al., 2017 [26]	PD; *N* = 17.	A = Cz (PMA-LL), C = SOA; DC: 0.06 mA/cm^2^; 30 min.	6 s; 2 weeks.	EV.1 = Pre EV.2 = Post EV.3 = 2 weeks post EV.4 = 6 weeks post	OFFLINE; Gradually off after 1 min.	BPG, UPDRS.	Conventional physiotherapy; 30 min.	SNS
Chang, Kim, & Park., 2015 [18]	Stroke; *N* = 12	A = APC of TA (PMA-affected LL), C = SOA-CL; A = 0.28 mA/cm^2^, C = 0.07 mA/cm^2^; 10 min.	10 s; 2 weeks.	EV.1 = pre EV.2 = 1 day post	ONLINE; Off after 15 s.	MEP of TA, BPG, FMA, MILS, FAC, BBS	Conventional physiotherapy; 30 min.	MEPs: ↓Latency (8.61%, *p* = 0.000) and ↑Width (50.4%, *p* = 0.048); ↑FMA (6.27%, *p* = 0.023) and ↑MILS (6.9%, *p* = 0.031)
Raithatha et al., 2016 [17]	SCI; *N* = 9	A = PMA-LL, C = SOA; A = 0.08 mA/cm^2^, C = 0.06 mA/cm^2^; 20 min.	36 s; 12 weeks.	EV.1 = pre EV.2 = post EV.3 = 4 weeks post	OFFLINE; Off after 30 s.	MMT, 6MWT, 10MWT, TUG, BBS, SCIM-III.	Exoskeleton robot; 1 h.	↑ MMT right at EV.2 (70.54%, *p* = 0.03) and EV.3 (91.5%, *p* = 0.01). MMT left: SNS ↑TUG in control group ↑6MWT in control group
Seo et al., 2017 [27]	Stroke; N = 9	A = lateral to CZ (PMI- affected LL), C = SOA-CL; DC: 0.06 mA/cm^2^; 20 min.	10 s; 2 weeks.	EV.1 = pre EV.2 = post EV.3 = 4 weeks post	OFFLINE; Off after 1 min.	FAC, 10MWT, 6MWT, BBS, FMA, MRCS, MEP (not in EV3).	Exoskeleton robot; 45 min.	↑FAC (44.5%, *p* = 0.031) at EV. 2 and ↑6MWT (60.35%, *p* = 0.038) at EV.3.
Costa-Ribeiro et al., 2017 [19]	PD; *N* = 11.	A = 2 cm anterior to CZ (PMA-LL), C = SOA-CL; CD: 0.06 mA/cm^2^; 13 min.	10 s; 4 weeks.	EV.1 = pre EV.2 = post EV.3 = 4 weeks post	OFFLINE; Off after 30 s.	10MWT, TUG, BPG, UPDRS part III (for motor deterioration), and UPDRS-Brad (bradykinesia), PDQ-39, BBS.	Visual cueing; 30 min.	SNS
Kumru et al., 2016 [16]	SCI; *N* = 12.	A = PMA-LL (vertex), C = SOA non-dominant; CD: 0.06 mA/cm^2^; 20 min.	20 s; 4 weeks.	EV.1 = pre EV.2 = post EV.3 = 4 weeks post	ONLINE; Off after 30 s.	LEMS (not at EV3), 10MWT, WISCI.	Exoskeleton robot; 30 min.	SNS

*10MWT* 10-m walk test, *6MWT* 6-min walking test, *A* anode, *APC* affected pre-central convolution, *BBS* Berg Balance scale, *BPG* biomechanical parameters of gait, *C* cathode, *CD* Current density, *PD* Parkinson’s disease, *CL* contra-lateral, *CZ* Cz area in accordance with the “International 10–20 System (EEC)”, *EOC* exterior occipital crest, *EV* evaluation, *FAC* functional ambulation categories, *FMA* Fugl-Meyer assessment, *LEMS* lower extremity motor score, *LL* lower limb, *MAS* modified Ashworth scale, *MEP* motor-evoked potential, *MILS* Motricity Index leg subscore, *MMT* manual muscle testing, *MRCS* Medical Research Council scale, *OFFLINE* tDCS applied before the intervention, *PDQ-39* Parkinson’s Disease Questionnaire-39, *PMA* primary motor area, *POMA* performance-oriented mobility assessment, *RMI* rivermead mobility index, *SCI* spinal cord injury, *SCIM-III* Spinal Cord Injury Measure, *SMA* supplementary motor area, *SNS* Statistically non-significant, *SOA* supra-orbital area, *TA* tibial anterior, *TIS* trunk impairment scale, *TUG* Timed Up and Go test, *UL-MT* upper limb motor task, *UNK* unknown, *UPDRS* unified Parkinson’s disease rating scale, *WISCI* walking index for spinal cord independence

### Participants

The samples that are part of this review were comprised of a total of 187 subjects with three different types of pathologies: 91 subjects with stroke [19, 23, 24, 26], of whom 54 were in the acute and 37 in the chronic phase (less or more than 6 months since the injury, respectively); 57 suffering from Parkinson’s disease [20, 21]; and 39 with spinal injury [17, 18]. Average age was 47.5–66.3 years and the ratio of women and men were 68 and 32%, respectively. Participants were included only when they completed the study.

### Stimulation patterns and parameters

In terms of electrode size, three studies employed 35 cm2 [21, 23, 24], Manji et al., 2018 [26] used 25 cm2 electrodes, Raithatha et al., 2016 [18] used 25 cm2 and 35 cm2 for the anode and cathode, respectively, and Chang, Kim, & Park., 2015 [19] employed 7.07 cm2 for the anode and 28.26 cm2 for the cathode. All the trials used anode stimulation with a single channel and two electrodes. Although the placement of electrodes varied among studies, a common application pattern was observed for all of them, where the active electrode (anode) was applied to the primary motor area, except for Manji et al., 2018 [26] that chose to apply it to the supplementary motor area.

In the included studies, the current intensities were 2 mA [17–21, 23], 1.5 mA [24], and 1 mA [26]. The current densities were 0.06 mA/cm2 [17, 18, 20, 21, 23] and 0.04 mA/cm2 [24, 26]. Raithatha et al., 2016 [18] applied a current density of 0.08 and 0.06 mA/cm2 and Chang, Kim, & Park., 2015 [19] used 0.28 and 0.07 mA/cm2 for the anode and cathode, respectively. The duration of tDCS sessions was one of the parameters showing more variability among studies ranging from 7 [24] to 30 min [21], although the most common length was 20 min [17, 18, 23, 26].

The protocol for applying sham tDCS varied among studies, although all followed some common pattern. The electrodes placement and stimulation parameters were equal to the experimental group, but some researchers raised and decreased the intensity to 0 mA in 1 min [21, 23], others in 30 [17, 18, 20] or 15 s [19], and others decided to keep the intensity at 0 mA the entire time [24].

In terms of the therapy for gait training that was combined with the tDCS, exoskeleton-robotic-assisted gait was used in six studies [17, 18, 23, 24, 26], followed by rehabilitation assisted by a physiotherapist in two studies [19, 21], and lastly, gait training via visual cueing in one study [20]. The duration of treatment for gait reeducation was a highly variable parameter, lasting 30 min in half of the studies [17, 19–21]. The application of tDCS combined with a specific technique for gait training was done simultaneously (online stimulation) in four studies [17, 19, 24, 26], whereas tDCS was applied before (offline stimulation) in the other four trials [18, 20, 21, 23].

There was great variability in the data in terms of total number of sessions and treatment duration. The overall number of sessions in the studies were 20 [17], 14 [26], 10 [19, 20, 23, 24], and 6 [21], with 10 sessions being observed most frequently. There seems to exist an agreement in terms of periods for performing the sessions since most authors conducted them in two [19, 21, 23, 24, 26] and four weeks [17, 20]. As an exception, the protocol by Raithatha et al., 2016 [18] comprised 36 sessions carried out throughout 12 weeks.

### Recorded variables and effect

In terms of the follow-up period, four studies evaluated the sample at four weeks [17, 18, 20, 23], one study at six weeks [24], and two studies assessed the sample immediately after the intervention but did not conduct any follow-up [19, 26].

Due to the large number of studied variables, we decided to group them in: i) functional variables, ii) clinical variables, and iii) biomechanical and neurophysiological variables. Although some of these measures do not directly evaluate gait function [motor score of lower limbs, motor evoked potentials (MEP), etc.], this decision was made to include them in the outcome of this review in order to offer more details about the global or indirect effect of tDCS on other approaches for gait training.

#### Functional variables

The most used scales were the 10-Meter Walk Test (10MWT) [17, 18, 20, 23, 24, 26] and Berg Balance Scale (BBS) [18–20, 23], although the latter was recorded only as a secondary variable. Only Manji et al., 2018 [26] reported a statistically significant improvement of ~ 10% in the 10MWT compared to sham stimulation. No study reported a significant difference on the BBS between the placebo and experimental groups.

Chang, Kim, & Park., 2015 [19] (among others) used the Fugl-Meyer Assessment (FMA) scale, whose index improved 6.27% in the experimental group compared to sham stimulation. On the other hand, Manji et al., 2018 [26] did not observe significant differences in the FMA between the sham and experimental groups, but an improvement of 5.29% was noted in the Timed Up and Go test (TUG). Costa-Ribeiro et al., 2017 [20] did not obtain an improvement in the TUG either. Furthermore, Raithatha et al., 2016 [18] observed an improvement in the sham group versus a non-significant improvement in the intervention group. However, the number of included subjects for this variable was only two and four for the control and intervention groups, respectively.

Seo et al., 2017 [23] documented a 44.5% post-intervention improvement in functional ambulation categories (FAC) for the intervention group compared to sham stimulation. In addition, a greater number of patients improved their score on this scale compared to those in the control group who had also experienced an improvement. A 60.35% improvement in the “6-Minute Walking Test” (6MWT) at four weeks post-intervention was also observed in the intervention group compared to sham stimulation. Nevertheless, Geroin et al., 2011 [24] did not find changes in the 6MWT and FAC; Chang, Kim, & Park., 2015 [19] also did not observe significant differences in the FAC compared to sham stimulation; and Raithatha et al., 2016 [18], similarly to the TUG variable, reported improved outcomes in the 6MWT in the placebo group (*n* = 2) compared to the experimental one (*n* = 6).

#### Clinical variables

To complement the analysis of gait rehabilitation, four trials also included clinical variables that could potentially effect or help to indirectly quantify it [18, 19, 23, 24]. The Motricity Index leg subscore (MILS), Medical Research Council scale (MRCS), and Manual Muscle Testing (MMT) were used to quantify the strength of the affected lower limb [18, 19, 23, 24] and the modified Ashworth scale (MAS) was used to assess its muscle tone [24]. The MILS was employed in two studies to assess the strength of the affected lower limb in patients with stroke, but only Chang, Kim, & Park, 2015 [19] obtained a 6.9% improvement in the experimental group compared to the sham group [19, 24]. The MRCS was used for the same purpose and no effect was observed [26]. Only Raithatha et al., 2016 [18] found a statistically significant improvement in the MMT in patients with spinal cord injuries (70% or 81% compared to the sham group depending on the follow-up), although their results were negative in terms of functional variables. Geroin et al., 2011 [24] utilized the MAS to assess the muscle tone of the affected lower limb (abductors, quadriceps, and plantar flexors) in patients with stroke and no intergroup differences were noted.

#### Biomechanical and neurophysiological variables

Geroin et al., 2011 [24] analyzed the cadence of stride, ratio of temporary symmetry (defined as the ratio between the oscillation time of the paralyzed and non-paralyzed limbs), and ratio between the single and double body-weight support on the lower limbs during the gait. Yotnuengnit et al., 2017 [21] assessed the ratio and cadence of stride. None of these studies observed statistically significant differences compared to sham stimulation.

The MEP of the tibial anterior [19] and abductor hallucis [23] muscles were assessed to complement the evaluation of the gait. Only the MEP for the anterior tibial muscle [19] showed an 8.61% decrease in latency and a 50.4% increase in range compared to the control group.

## Discussion

Based on the results observed in this systematic review, where half the included trials did not report improvements in any variable in the experimental group compared to sham stimulation [17, 20, 21, 23], it can be deduced that there are no conclusive results supporting the notion that tDCS enhances the effect of current methods for gait rehabilitation in patients with neurological disorders. The large variability observed in the stimulation patterns and parameters, as well as in registered variables, hinders the analysis and comparison of outcomes in order to determine, in an objective way, the actual effectiveness of the technique and optimal parameters for its application.

The anodic stimulation of the primary motor area of lower limbs was the most common protocol for all studies; the single exception was the study by Manji et al., 2018 [26] that stimulated the supplementary motor area and was the only one reporting an improvement in the 10MWT and TUG variables compared to the sham group. However, other trials stimulating the primary motor area showed positive effects on outcome variables such as the 6MWT [23], functional ambulation [23], muscle strength [18], and mobility and functionality of lower limbs [19]. Hence, further research is required to optimize the stimulation areas, including evaluation of the dorsolateral prefrontal cortex, which has shown to increase upper limbs strength [22, 43], or the area for upper-limb cortical representation, which has revealed an increase in the strength of the knee extensors [44] in healthy volunteers. The deep representation of lower limbs in the motor cortex within the interhemispheric fissure can be a limitation for the effectiveness of tDCS on activities like walking. New paradigms should be studied for gait rehabilitation, for example, high-density stimulation that achieves a greater focalization of the current [45].

The stimulation intensity is another factor that could be key for determining the effectiveness of the technique [46]. Although the difference in the applied intensity was not substantial among the included studies, the electrodes size varied greatly, which significantly alters current density. Studies on peripheral stimulation [47] have recommended expressing the stimulation intensity in terms of current density in order to avoid mistakes and allow for comparison among trials.

In spite of the limited data obtained from the assessed studies, the number of sessions and stimulation duration appear to be a key factor for determining the intervention effectiveness. On the whole, it can be noticed how a greater number of sessions and longer session times increase intervention effectiveness [18, 19, 23, 26]. However, Kumru et al. 2016 [17] applied 20 sessions and reported a lack of effect, and the trial by Raithatha et al., 2016 [18] produced conflicting outcomes where, after applying 36 sessions, an improvement in strength (*n* = 9) was observed in the intervention group compared to the sham group; however, both the 6MWT (*n* = 6) and TUG (*n* = 4) improved more in the control group (*n* = 2) compared to the tDCS intervention. Future trials should investigate the specific effects of stimulation programs comprising > 10 sessions.

In terms of the duration of the effect, of the four trials that reported improved outcomes in at least one variable compared to the sham group [18, 19, 23, 26], only two carried out a follow-up once the program finished, during which they observed that the effect lasted up to one month [18, 23]. Patients responding positively to the intervention appeared to achieve a relatively long-lasting effect that should be assessed with more prolonged follow-up periods. On the other hand, other factors like the combined gait rehabilitation strategy or its duration did not yield relevant outcomes in this review.

One aspect that could be of importance in the paradigm of combining tDCS with other therapies is whether the treatments are applied simultaneously (online stimulation) or whether the therapy is applied following stimulation (offline stimulation). Of the eight analyzed studies, four applied online stimulation [17, 19, 24, 26], half of which showed its effectiveness [19, 26] and the other half did not [17, 24]. Since there was no trial on the various applications of tDCS that performed a direct comparison of online and offline stimulation, specific studies should be conducted along these lines to optimize the potential therapeutic use of tDCS when combined with other treatments.

Although none of the assessed studies reported adverse effects, this was not investigated in a systematic way in any study. Several trials outlining minor and transient adverse effects have supported the safety of the technique [48]. Additionally, the observed adverse effects were found in similar proportions to those reported in subjects receiving placebo stimulation [49].

The main limitation of this systematic review is the restricted number of trials conducted for a great diversity of parameters, application patterns, and assessed variables. Also, drawing conclusions is complicated in sight of the various studied pathologies. Overall, the three trials performed in patients with stroke, with a minimal stimulation lasting 10 min, showed improvements in at least one variable of gait or functionality [19, 23, 26]. However, these outcomes must be replicated in future research to state a conclusion. On the other hand, we attempted to select studies with at least five stimulation sessions and five patients per group in order to obtain more reliable, clinically applicable results. Nevertheless, such rigor in the selection criteria may have discarded possible articles of relevance from a scientific point of view and masked the results.

## Conclusions

In sight of the analyzed outcomes, there are no conclusive results to support a role for tDCS in enhancing the effect of other gait rehabilitation strategies. However, the great variability of assessed parameters and protocols, as well as pathologies and obtained outcomes, highlights the need for further research that investigate how to optimize tDCS as a therapeutic tool to improve the effect of the various existing gait training techniques in patients with neurological disorders.

## Data Availability

The data collected in this study are available from the corresponding author on reasonable request. All primary data were extracted from the referenced sources.

## Abbreviations

10MWT: 10-m walk test
6MWT: 6-min walking test
FAC: Functional ambulation categories
FMA: Fugl-Meyer assessment
MAS: Modified Ashworth scale
MEP: Motor-evoked potential
MILS: Motricity Index leg subscore
MMT: Manual muscle testing
MRCS: Medical Research Council scale
PEDro: Physiotherapy Evidence Database
tDCs: Transcranial direct current stimulation
TUG: Timed Up and Go test
